# Novel prognostic signature for hepatocellular carcinoma using a comprehensive machine learning framework to predict prognosis and guide treatment

**DOI:** 10.3389/fimmu.2024.1454977

**Published:** 2024-09-24

**Authors:** Shengzhou Zheng, Zhixiong Su, Yufang He, Lijie You, Guifeng Zhang, Jingbo Chen, Lihu Lu, Zhenhua Liu

**Affiliations:** ^1^ Department of Emergency, Fujian Medical University Union Hospital, Fuzhou, China; ^2^ Department of Oncology, Shengli Clinical Medical College of Fujian Medical University, Fujian Provincial Hospital, Fuzhou University Affiliated Provincial Hospital, Fuzhou, Fujian, China; ^3^ Department of Radiation Oncology, Fujian Medical University Union Hospital, Fuzhou, China

**Keywords:** hepatocellular carcinoma, prognosis signature, treatment, machine learning framework, TCGA

## Abstract

**Background:**

Hepatocellular carcinoma (HCC) is highly aggressive, with delayed diagnosis, poor prognosis, and a lack of comprehensive and accurate prognostic models to assist clinicians. This study aimed to construct an HCC prognosis-related gene signature (HPRGS) and explore its clinical application value.

**Methods:**

TCGA-LIHC cohort was used for training, and the LIRI-JP cohort and HCC cDNA microarray were used for validation. Machine learning algorithms constructed a prognostic gene label for HCC. Kaplan–Meier (K-M), ROC curve, multiple analyses, algorithms, and online databases were used to analyze differences between high- and low-risk populations. A nomogram was constructed to facilitate clinical application.

**Results:**

We identified 119 differential genes based on transcriptome sequencing data from five independent HCC cohorts, and 53 of these genes were associated with overall survival (OS). Using 101 machine learning algorithms, the 10 most prognostic genes were selected. We constructed an HCC HPRGS with four genes (SOCS2, LCAT, ECT2, and TMEM106C). Good predictive performance of the HPRGS was confirmed by ROC, C-index, and K-M curves. Mutation analysis showed significant differences between the low- and high-risk patients. The low-risk group had a higher response to transcatheter arterial chemoembolization (TACE) and immunotherapy. Treatment response of high- and low-risk groups to small-molecule drugs was predicted. Linifanib was a potential drug for high-risk populations. Multivariate analysis confirmed that HPRGS were independent prognostic factors in TCGA-LIHC. A nomogram provided a clinical practice reference.

**Conclusion:**

We constructed an HPRGS for HCC, which can accurately predict OS and guide the treatment decisions for patients with HCC.

## 1 Introduction

Hepatocellular carcinoma (HCC) is a major challenge in global health, ranking sixth among common malignancies and third in terms of tumor mortality. HCC is the most common pathological type of liver cancer, accounting for approximately 80% of primary liver malignancies (1). Previous studies have shown that only 36% of liver cancer cases in China are diagnosed early and meet the criteria for radical treatment. Among the remaining cases, 9% and 55% of patients are in the intermediate and advanced stages, respectively (2).

Radical treatment is recommended for patients with early-stage HCC, including surgical treatment (resection and liver transplantation) and local-regional surgery (radiofrequency ablation) (3). Patients with intermediate-stage HCC are recommended to undergo transarterial local-regional therapy (4). For patients with advanced HCC, comprehensive systemic treatment based on chemotherapy, targeted therapy, and immunotherapy is the main treatment strategy (4). In recent years, because of the rapid development of targeted therapy and immunotherapy, significant changes have occurred in the treatment and prognosis of advanced HCC. The results of two clinical trials, SHARP and ORIENTAL, both showed that sorafenib can prolong the survival of patients with advanced HCC. Therefore, sorafenib has been approved as a first-line treatment for patients with advanced HCC since 2007 (5, 6). The REFLECT study showed that lenvatinib is not inferior to sorafenib, and the median overall survival (OS) of lenvatinib and sorafenib is similar, but the objective response rate (ORR) and progression-free survival of lenvatinib are higher (7). The IMbrave150 study showed that the combination of atezolizumab and bevacizumab is more effective in improving OS, and was approved for first-line treatment of patients with advanced HCC in China in 2021 (8). There are currently several ongoing studies on immunotherapy-based combination therapy. However, because of the high heterogeneity of liver cancer and other factors, the annual recurrence rate of HCC is as high as 15%–20%. Currently, the accuracy of biomarkers, such as alpha-fetoprotein (AFP), programmed cell death 1 ligand 1 (PD-L1), and tumor mutation burden, is still insufficient to guide the precision diagnosis and treatment of HCC. Therefore, it is of great significance to explore more accurate and effective biomarkers for optimizing the clinical diagnosis and treatment of patients with HCC and improving prognosis.

The pathogenesis and progression of malignant tumors cover a wide range of complex multistage processes. In the past few decades, thanks to breakthroughs in the fields of genomics, transcriptomics, proteomics, and metabolomics, significant progress has been made in precision medicine strategies for cancer diagnosis and treatment (9, 10). The occurrence and development of liver cancer have been confirmed to be driven by molecular variations at the genetic and epigenetic levels (11). In the field of HCC, the application of next-generation sequencing technology provides important evidence for revealing molecular changes (12–14). Given the differences in molecular biological characteristics, even patients at the same stage of the disease may have similar tumor morphology and clinical manifestations but still respond differently to the same treatment strategy. Therefore, the increasing use of genome analysis technology to deeply explore tumor biology and assist in the development of individualized treatment plans for patients with HCC could improve their prognosis (15). Prediction models based on cancer gene expression are one of the important research directions of gene transcriptomics in cancer research. In several cancers, such prediction models have been successfully applied to molecular subtypes, prognosis, and treatment prediction in clinical practice (16). However, previous studies have developed many prediction models related to HCC and demonstrated relatively good performance in some cohorts. However, considering that these prediction models are constructed based on messenger ribonucleic acid (mRNA), microRNA (miRNA), or long noncoding ribonucleic acid (lncRNA) in specific pathways (such as immunogenic cell death, necroptosis, ferroptosis, and cuproptosis), the utilization of data is insufficient (17–19). In addition, because of the uniqueness and inappropriateness of the selected modeling methods, prognostic models based on multiple genes have significant shortcomings, which limit their widespread clinical application (20).

Therefore, in this study, we aimed to establish a comprehensive and accurate prognostic signature for HCC by integrating multiple machine learning methods and applying multiple independent cohort datasets to construct and validate an HCC prognosis-related gene signature (HPRGS). The characteristics and clinical application value of this signature will be analyzed to provide a reference for the clinical diagnosis and treatment of HCC.

## 2 Materials and methods

### 2.1 Data acquisition

We downloaded 7 cohorts (GSE13845, GSE25097, GSE84402, GSE174570, GSE54236, GSE14520 and GSE104580) from the GEO database (https://www.ncbi.nlm.nih.gov/geo/), and the data were subjected to log2 transformation for subsequent analysis (21). The TCGA-LIHC and LIRI-JP cohorts were obtained from the Cancer Genome Atlas (TCGA; https://portal.gdc.cancer.gov/) and the ICGC database (https://dcc.icgc.org/releases), respectively. Additionally, somatic mutation data for the TCGA-LIHC cohort was obtained from the TCGA database. Only patients with complete clinical follow-up information and survival data were included in the TCGA-LIHC and LIRI-JP cohorts. After screening, 365 patients with hepatocellular carcinoma were retained in the TCGA-LIHC cohort, and 231 patients were retained in the LIRI-JP cohort. To improve comparability between cohorts, the RNA-sequencing (RNA-seq) was converted to transcripts per million (TPM) forma. Then, the batch correction was performed on the TCGA-LIHC cohort and the LIRI-JP cohort using the “Combat “ method from the “sva” R package.

In addition, the HCC cDNA microarray was purchased from Shanghai Outdo Biotech Co.,Ltd (Shanghai, China) with 87 samples (including 21 normal liver samples and 66 HCC samples, Ethics No.SHYJS-CP-1707015). The clinical characteristics of these cohorts were integrated in **Table 1**.

**Table 1:** The clinical characteristics of TCGA-LIHC, LIRI-JP and HCC cDNA microarry cohorts.

Characteristics	TCGA-LIHC	LIRI-JP	HCC cDNA microarry
n	365	231	66
**Age, n (%)**			
>60	192 (52.6%)	182 (78.8%)	23 (34.8%)
<=60	173 (47.4%)	49 (21.2%)	43 (65.2%)
**Gender, n (%)**			
MALE	246 (67.4%)	170 (73.6%)	57 (86.4%)
FEMALE	119 (32.6%)	61 (26.4%)	9 (13.6%)
**Grade, n (%)**			
G1-2	230 (63.0%)	-	64 (97.0%)
G3-4	130 (35.6%)	-	2 (3.0%)
unknow	5 (1.4%)	-	-
**Stage, n (%)**			
Stage I-II	254 (69.6%)	141 (61.0%)	56 (84.8%)
Stage III-IV	87 (23.8%)	90 (39.0%)	10 (15.2%)
unknow	24(6.6%)	-	-

### 2.2 Quantitative reverse transcription PCR

Relative quantitation was determined by quantitative reverse transcription polymerase chain reaction (qRT-PCR; SuperScript IV Reverse Transcriptase 18090010; Thermo Fisher, United States). The amplification reactions were performed as described previously (22). LCAT-specific primers were: forward primer, 5’-GTGACTTCCAACGCTTCTT-3’ and reverse primer, 5’-TCATAGAGCACACCCACAG-3’. SOCS2-specific primers were: forward primer, 5’-CCTTGCCTTCTTAGGTTCTT-3’ and reverse primer, 5’-CTTGGTTCCTTCCCACTT-3’. ECT2-specific primers were: forward primer, 5’-TGTAGTCACGGACTTTCAGGA-3’ and reverse primer, 5’-GTACAATACAACGGGCGACAT-3’. TMEM106C-specific primers were: forward primer, 5’-TTCACCGGGAGAGATAGCATC-3’ and reverse primer, 5’-AAGGACTGAATGCGGAAACAG-3’.

### 2.3 Differential gene analysis

The “Limma” package (23) in R software was used to screen for differentially expressed genes (DEGs) between normal tissue and HCC tissue in the TCGA-LIHC, GSE13845, GSE25097, GSE84402, and GSE174570 cohorts. Multiple testing with a FDR<0.05 and |log2Foldchange|≥ 1 were used as screening criteria.

### 2.4 The establishment of HPRGS

This study followed the following steps to construct HPRGS:

Firstly, the “survival” package in R software was used to perform univariate Cox regression analysis to screen for DEGs with potential prognostic value in the gene expression profiles of patients in the TCGA-LIHC cohort.

The TCGA-LIHC cohort was used as the training cohort, and the LIRI-JP cohort was used as the validation cohort. In the training cohort, 101 combinations of 10 algorithms were used for variable selection based on a ten-fold cross-validation framework. The 10 machine learning algorithms included Least Absolute Shrinkage and Selection Operator Regression Algorithm (Lasso, “glmnet” R package), Ridge Regression Algorithm (Ridge, “glmnet” R package), Stepwise Cox Proportional Hazards Regression Algorithm (stepwise Cox, “stepwise” R package), CoxBoost Algorithm (CoxBoost, “CoxBoost” R package), Random Survival Forest Algorithm (RSF, “RandomForestSRC” R package), Elastic Net Regression Algorithm (Enet, “glmnet” R package), Partial Least Squares Regression To Cox Models Algorithm (plsRcox, “plsRcox” R package), Supervised Principal Components for regression Algorithm (SuperPC, “superpc” R package), Gradient Boosting Machine Algorithm (GBM, “gbm” R package), and Survival support vector machines Algorithm (survival-SVM, “survivalsvm” R package). For each model, we calculated the C-index on the training and validation sets. Then, we ranked the predictive performance of the models based on the average C-index. Finally, we selected a robust combination of algorithms.

Finally, we performed multivariate Cox regression analysis to further screen genes to construct HPRGS and established the risk score for quantification using the gene expression values and coefficients. The scoring formula is as follows:


$$\text{Risk score}=\sum_{\text{i}=1}^{\text{n}}\text{Coef}\left(\text{i}\right)\times \text{x}\left(\text{i}\right)$$

Based on the scoring formula, we determined the risk score for each HCC patient in the TCGA-LIHC, LIRI-JP and HCC cDNA microarray cohorts and classified them into high-risk and low-risk groups based on the median risk score (24).

### 2.5 The evaluation of HPRGS

The “survival” package in R software was used to conduct survival analysis to investigate whether there was a significant difference in OS between low- and high-risk groups. The results were visualized using the “survminer” package in R software. Additionally, the “timeROC” package in R software was used to perform receiver operating characteristic (ROC) curve analysis to assess the sensitivity and specificity of risk scores in predicting OS in HCC patients.

### 2.6 Gene function analysis

The “org.Hs.eg.db, clusterProfiler, GOplot” packages in R software were used to perform gene ontology (GO) functional annotation and Kyoto Encyclopedia of Genes and Genomes (KEGG) pathway analysis for prognostic-related differentially expressed genes based on the gene set files “c5.go.v7.4.symbols.gmt” and “c2.cp.kegg.v7.4.symbols.gmt” from the Molecular signatures database (MSigDB). The enrichment of prognostic-related DEGs in GO and KEGG was calculated, and signaling pathways with multiple testing P<0.05 were obtained, displaying the biological processes (BP), cellular components (CC), molecular functions (MF), and pathways involved in the differentially expressed genes. Subsequently, gene set enrichment analysis (GSEA) was conducted to explore the biological differences between the high- and low-risk groups. Additionally, single-sample gene set enrichment analysis (ssGSEA) based on the “c1.hallmark.v7.4.symbols.gmt” gene set file from MSigDB was performed using the “GSVA” package in R software to calculate scores for 50 hallmark pathways. Then, the “limma” package in R software was used to analyze the significantly different pathways between the high-risk and low-risk groups, and the “Hmisc” package was used to calculate the correlation between the 50 hallmark pathways and risk scores (25). Furthermore, the “survival” package in R software was used to analyze the relationship between each pathway and OS.

### 2.7 Genomic variation analysis

Mutant-allele tumor heterogeneity (MATH) is a method that quantitatively measures intra-tumor heterogeneity (ITH) based on the distribution of mutant alleles. MATH scores are obtained through whole-exome sequencing of tumor and matched normal samples, providing a measurable and quantitative assessment of ITH, and the higher MATH scores were associated with more severe ITH (26). The prognostic significance of MATH has been explored in head and neck cancer, colorectal cancer, and breast cancer (27). In this study, the MATH algorithm was used to evaluate ITH in HCC patients. Additionally, to investigate somatic mutations associated with HCC, mutation waterfall plots were generated for HCC patients in the high- and low-risk groups using the “maftools” package in R software.

### 2.8 Analysis of immune microenvironment

To examine the association between risk scores and immune cell infiltration in the HCC tumor microenvironment, this study first used the ESTIMATE algorithm to calculate the abundance of stromal cells and immune cells, as well as tumor purity. Then, the CIBERSORT deconvolution algorithm was used to quantify the infiltration of 22 immune cell types (28). The anticancer immune cycle is an important component of tumor immunotherapy, consisting of seven key steps: cancer antigen release (step 1), cancer antigen presentation (step 2), priming and activation (step 3), immune cell trafficking to the tumor (step 4), immune cell infiltration into the tumor (step 5), T-cell recognition of cancer cells (step 6), and killing of cancer cells (step 7). These seven steps together constitute the anticancer immune cycle. The activity scores of the seven anticancer immune steps for TCGA-LIHC samples were obtained from the Tracking Tumor Immunophenotype (TIP) platform (http://biocc.hrbmu.edu.cn/TIP/index.jsp) (29).

### 2.9 Drug sensitivity analysis

The “oncoPredict” package of R software was used to predict the chemotherapy sensitivity of HCC patients with different HPRGS based on the Genomics of Drug Sensitivity in Cancer (GDSC) database (30). The “oncoPredict” package of R software fits the tissue gene expression profiles of patients with the expression profiles of cancer cell lines to calculate the half maximal inhibitory concentration (IC50). Drugs with significant differences in IC50 between the high- and the low-risk group were screened.

### 2.10 Prediction of immunotherapy response

The Tumor Immune Dysfunction and Exclusion (TIDE, http://tide.dfci.harvard.edu/) is used to assess the possibility of tumor immune evasion in the gene expression profiles of tumor samples (31). The Immunophenoscore (IPS) algorithm, which uses machine learning methods to calculate the IPS score based on unbiased gene expression of representative cell types (32). The IPS scores of TCGA-LIHC patient samples were obtained from the Cancer Immunome Atlas (TCIA, https://tcia.at/home) database. Based on the IPS score, immune checkpoint inhibitors (ICI) treatment was divided into the following four categories (1): CTLA4+/PD1+ treatment, (2) CTLA4+/PD1- treatment, (3) CTLA4-/PD1+ treatment, (4) CTLA4-/PD1- treatment.

### 2.11 Exploration of potential therapeutic drugs

We explored potential therapeutic drugs for patients with high-risk HCC based on previous protocols (33). First, we obtained drug sensitivity data of cancer cell lines (CCLs) from Cancer Therapeutics Response Portal (CTRP, https://portals.broadinstitute.org/ctrp) website and Profiling relative Inhibition Simultaneously In Mixtures (PRISM, https://depmap.org/portal/prism/) drug reuse resources, and obtained CCLs expression data from Cancer Cell Line Encyclopedia (CCLE) database. CTRP contains sensitivity data of 481 compounds to 835 CCLs, and PRISM contains sensitivity data of 1448 compounds to 482 CCLs. Both cohorts provide dose-response AUC values as a measure of drug sensitivity, with lower AUC values indicating increased sensitivity to treatment. In addition, as a first-line chemotherapeutic drug for HCC, we further selected gemcitabine to verify the scientificity and rigor of this method. We used the “Hmisc” package of R software to perform correlation analysis to further screen compounds with negative correlation coefficients between AUC values and HPRGS (setting the threshold R<-0.3). Then, we performed differential analysis of the selected drugs between high- and low- risk groups, and selected compounds with significantly lower AUC values in the high risk group. Connectivity Map (CMap, https://clue.io/) is a publicly available web tool for exploring candidate compounds that may target HPRGS-related pathways based on gene expression profiles (34, 35). Based on differential expression analysis and correlation analysis, we used CMap to identify potential compounds in HCC to further validate the results obtained from CTRP and PRISM databases.

### 2.12 Construction and evaluation of nomogram

Firstly, univariate and multivariate Cox regression analysis were used to evaluate the correlation with OS between different clinicopathological factors and HPRGS in TCGA-LIHC cohort. In both univariate and multivariate Cox analysis, factors with P<0.05 were determined to have an independent correlation with OS in patients with HCC. The “RMS” package of R software was used to construct the nomogram. The nomogram was constructed by combining HPRGS with other independent prognostic factors. To evaluate the predictive effect of the nomogram on patient prognosis, this study used the “RMS” package of R software to predict the prognostic calibration curves for 1-, 2-, and 3-year survival rates in the TCGA-LIHC cohort. Then, the timeROC R package was used for 1-, 2-, and 3-year ROC curve analysis to evaluate the sensitivity and specificity of the nomogram in predicting OS in HCC patients.

### 2.13 Statistical analysis

All statistical analyses in this study were performed using R studio (versions: R 4.1.0 and R 4.3.0). Continuous variables were analyzed using the Wilcoxon rank-sum test. Categorical variables were statistically compared using the chi-square test. Unless otherwise specified, statistical significance was set at P <0.05.

## 3 Results

### 3.1 Identification of prognostic genes and functional annotations related to HCC

**Figure 1:**
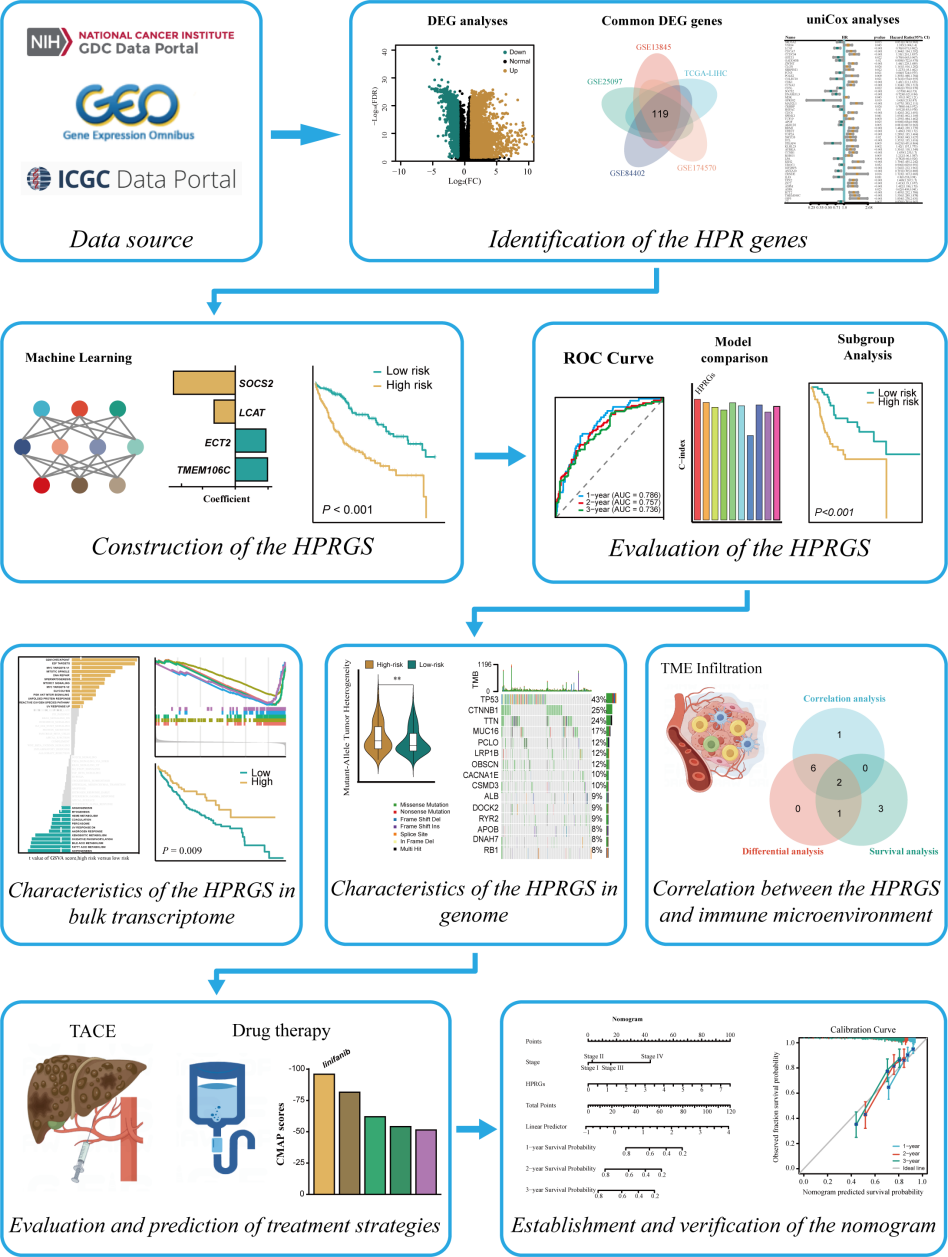
The entire analytical process of the study.

**Figure 2:**
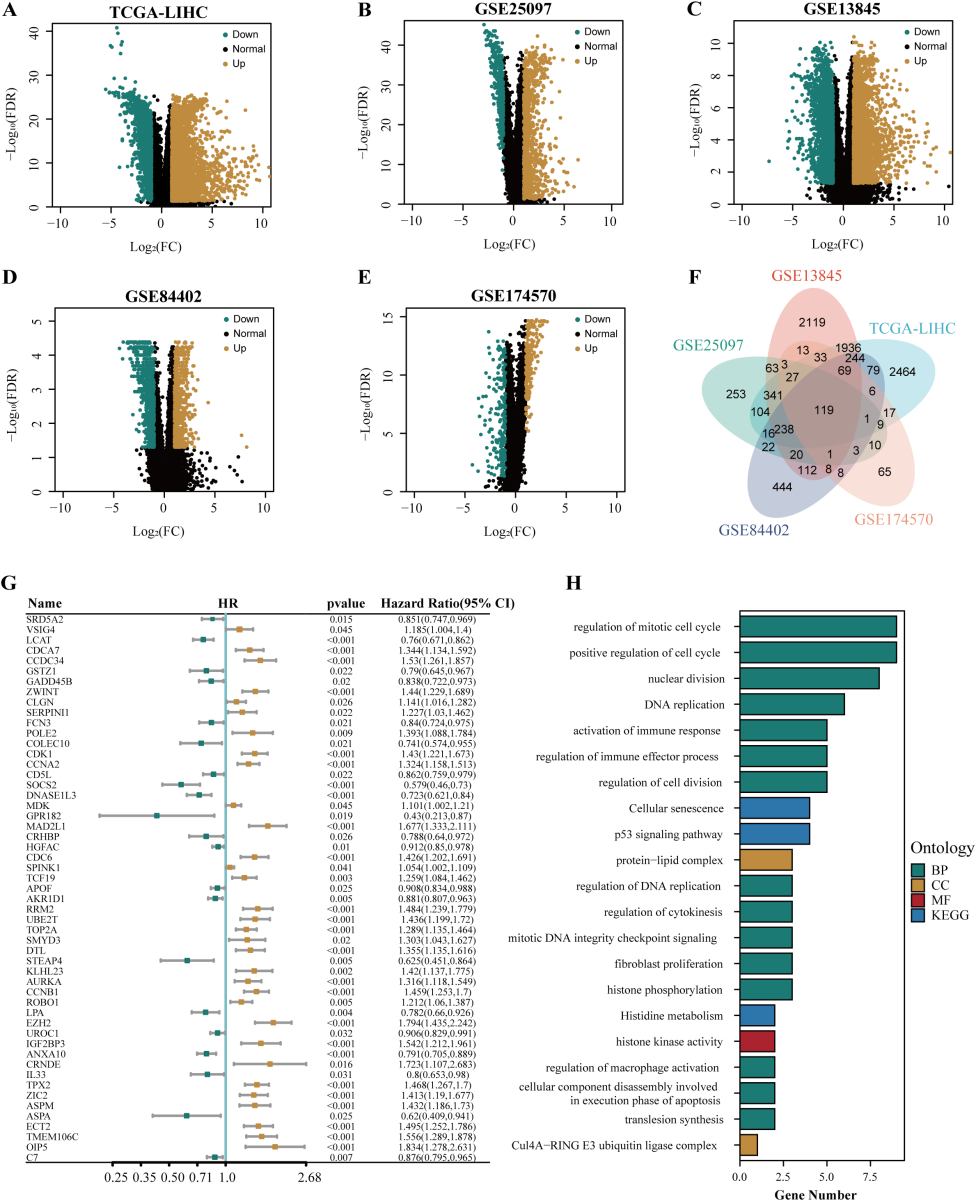
The identification and functional annotation of prognostic genes in HCC. The volcano plots showed the differentially expressed genes in TCGA-LIHC cohort **(A)**, GSE25097 cohort **(B)**, GSE13845 cohort **(C)**, GSE84402 cohort **(D)**, and GSE174570 cohort **(E)**. **(F)** The venn plot illustrated the overlapping differentially expressed genes in multiple cohorts, including TCGA-LIHC, GSE25097, GSE13845, GSE84402, and GSE174570. **(G)** The forest plot depicted the common differentially expressed genes associated with the prognosis of HCC. **(H)** The results of the enrichment analysis for GO and KEGG were presented.

The flow chart of this study is shown in **Figure 1**. To identify the differential genes in HCC, differential expression analysis was performed on normal and HCC tissues in TCGA-LIHC, GSE13845, GSE25097, GSE84402, and GSE174570 cohorts. The specific results were 5,703 differential genes screened from TCGA-LIHC cohort (**Figure 2A**), 5,346 from the GSE25097 cohort (**Figure 2B**), 1,230 from the GSE13845 cohort (**Figure 2C**), 1,403 from the GSE84402 cohort (**Figure 2D**), and 399 from the GSE174570 cohort (**Figure 2E**) (**Supplementary Table 1**). Further intersection analysis of the differential genes resulted in 119 common differentially expressed genes (DEGs) (**Figure 2F**). Through univariate Cox analysis of these 119 DEGs, 53 genes with prognostic significance were selected for subsequent analysis (all P<0.05, **Figure 2G**).

To explore the function of prognosis-related differential genes, we performed GO and KEGG enrichment analysis on the 53 genes with prognostic significance. GO enrichment analysis showed that these genes were highly enriched in DNA replication regulation, apoptotic nuclear changes, cell component disassembly involved in apoptotic execution, macrophage activation regulation, positive regulation of phagocytosis, steroid catabolism, immune effect process regulation, and immune response activation. KEGG enrichment analysis showed that these genes were highly enriched in the P53 signaling pathway, cellular senescence, histidine metabolism, and cell cycle pathways (**Figure 2H**).

### 3.2 Construction of HCC HPRGS

**Figure 3:**
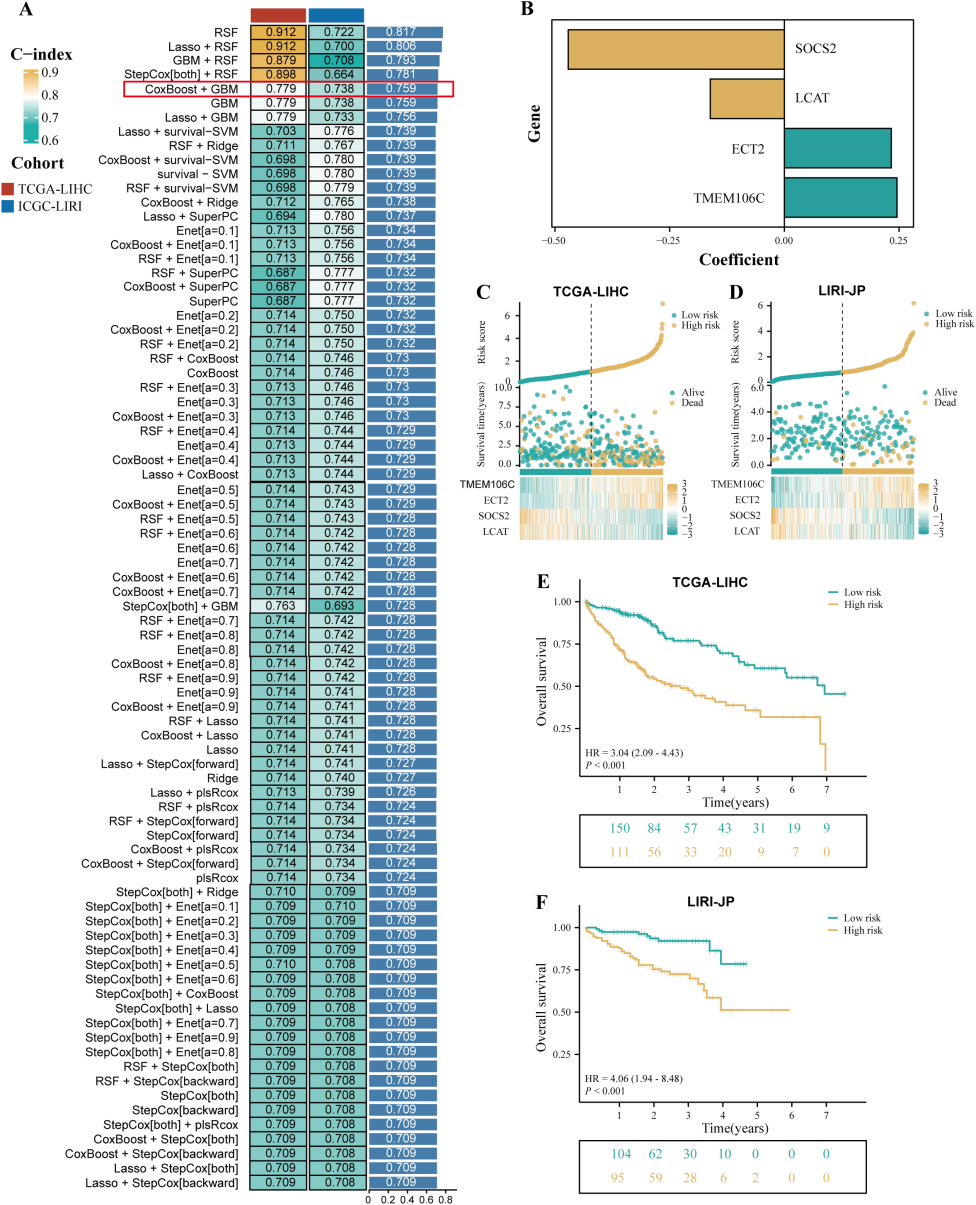
The development of a prognostic signature for HCC. **(A)** A total of 101 kinds of prediction signatures via a ten-fold cross-validation framework and further calculated the C-index of each signatures across all validation datasets. **(B)** The barplot showed the regression coefficients of 4 genes obtained in multivariate Cox regression. The risk factor plot of the TCGA-LIHC cohort **(C)** and the LIRI-JP cohort **(D)**. The Kaplan–Meier curves of OS according to the HPRGS in the TCGA-LIHC cohort **(E)** and LIRI-JP cohort **(F)**.

TCGA-LIHC cohort was used as the training cohort, and the LIRI-JP cohort was used as the validation cohort. During the training process, 101 prediction models were combined using a 10-fold cross-validation framework, and the C-index was calculated for all training and validation cohorts (**Figure 3A**). Among the models constructed using 101 machine learning algorithms, the average C-index evaluation showed that although the first four prediction models performed well in the training cohort, their performance in the validation cohort was significantly different, indicating possible overfitting. Therefore, these models that overfit the training cohort were excluded from further selection. Subsequently, the CoxBoost+GBM model was selected because it exhibited good predictive ability in both the validation cohort and the training cohort (C-index > 0.7). The model included 10 genes (LCAT, CCDC34, SOCS2, EZH2, ANXA10, TPX2, ZIC2, ECT2, TMEM106C, and VSIG4), and further model construction was performed using multivariable Cox analysis to identify four key genes (SOCS2, LCAT, ECT2, and TMEM106C). Subsequently, the expression levels of these four genes were weighted using regression coefficients from the Cox model to calculate the risk score for each patient (**Figure 3B**). We defined this signature as HPRGS, with the formula: HPRGS = 0.245484853986847*TMEM106C gene expression + 0.233006449350621*ECT2 gene expression - 0.4709503811778*SOCS2 gene expression - 0.161437679389049*LCAT gene expression. Based on the median value of HPRGS, all patients in the training cohort and validation cohort were divided into high- and low-risk groups. In both the validation cohort and the training cohort, the number of deaths increased gradually with increasing HPRGS scores (**Figures 3C, D**). Further survival analysis showed that in the training cohort, the OS of patients in the high-risk group was significantly lower than that in the low-risk group (P<0.001, HR=3.04, **Figure 3E**), and consistent results were observed in the validation cohort (P<0.001, HR=4.06, **Figure 3F**).

### 3.3 Evaluation of HCC HPRGS

**Figure 4:**
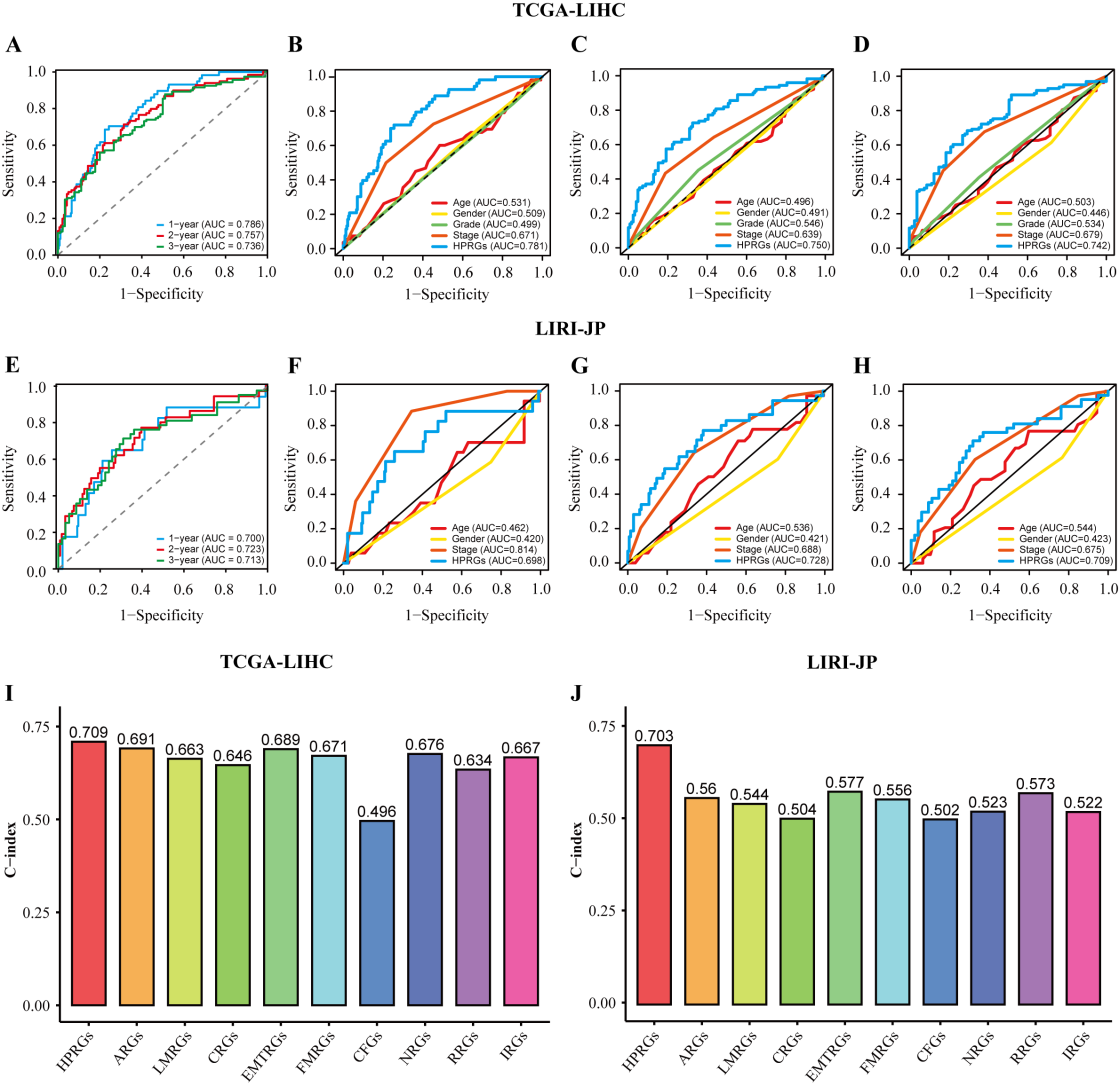
The assessment of HPRGS. ROC curves showed the specificity and sensitivity of HPRGS and clinical characteristics in predicting 1, 2, and 3-year OS in the TCGA-LIHC cohort **(A–D)** and LIRI-JP cohort **(E–H)**. C-index of 10 prognostic signatures in TCGA-LIHC **(I)** and LIRI-JP **(J)**.

To evaluate the prognostic effectiveness of HPRGS, ROC curve analysis was conducted. In TCGA-LIHC training cohort, the AUCs of HPRGS reached 0.786, 0.757, and 0.736 at 1-, 2-, and 3-years, respectively, and in the LIRI-JP validation cohort, they reached 0.700, 0.723, and 0.713, respectively (**Figures 4A**, **E**). Then, the clinical information (including age, sex, grade, and stage) for each patient in the training cohort was compared with their corresponding HPRGS through ROC curve analysis. The results showed that the prognostic effectiveness of HPRGS at 1-, 2-, and 3-years was better than other clinical characteristics (**Figures 4B–D**), and consistent results were obtained in the validation cohort (**Figures 4F–H**). Furthermore, they were compared with nine published HCC prognostic models, including the anoikis-related genes signature (ARG) (36), lactic acid metabolism-related gene signature (LMRG) (37), cuproptosis-related gene signature (CRG) (19), epithelial-mesenchymal transition-related gene signature (EMTRG) (38), fatty acid metabolism gene signature (FMRG) (39), cancer-associated fibroblast signature (CFG) (40), necroptosis-related gene signature (NRG) [21], inflammatory response-related gene signature (RRG) (41), and immunotherapy-related gene signature (IRG) (17). We used the C-index to evaluate the predictive ability of the models, and the results showed that HPRGS had the highest C-index in both the training and validation cohorts (**Figures 4I, J**). These results indicated that the HPRGS has good accuracy in predicting the prognosis of patients with HCC.

In addition, since clinical features are commonly used in clinical practice to assess the prognosis of patients with HCC, subgroup analysis was performed on high- and low-risk groups of patients with HCC based on age (**Supplementary Figures 1A, B**), sex (**Supplementary Figures 1C, D**), pathological grade (**Supplementary Figures 1E, F**), and pathological stage (**Supplementary Figures 1G, H**) in the training cohort. Similar to the results in the training and validation cohorts, patients with HCC in the high-risk group with different clinical characteristics exhibited poorer survival rates than the low-risk group (all P<0.05). In addition, ROC curve analysis showed that HPRGS had comparable predictive ability at 1-, 2-, and 3-years for patients with different clinical characteristics.

### 3.4 Biological functions of patients in high- and low-risk groups

**Figure 5:**
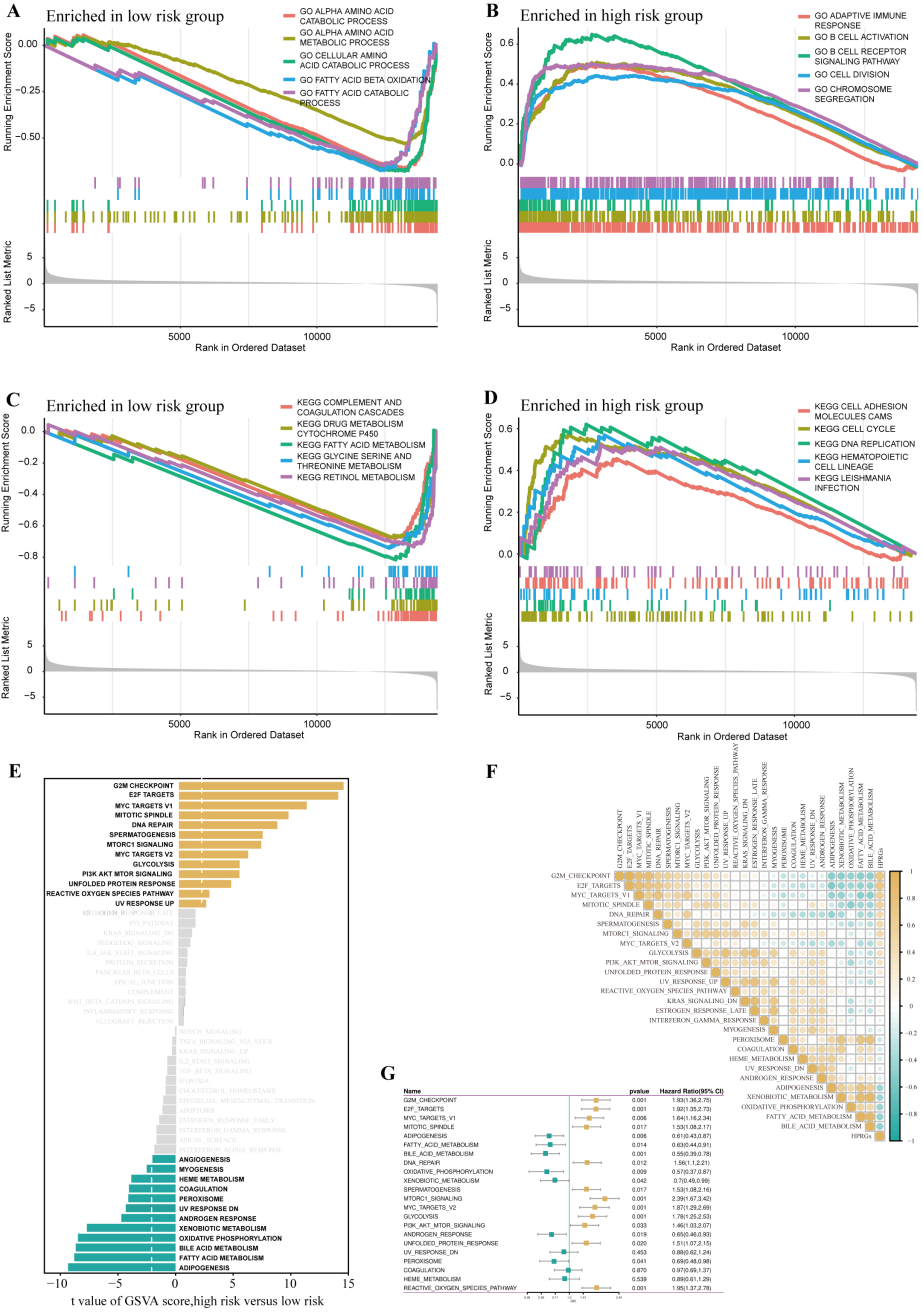
Disparities in biological functionality between high- and low-risk groups. GO terms enriched in the low- **(A)** and high-risk group **(B)** by GSEA analysis. KEGG terms enriched in the low- **(C)** and high-risk group **(D)** by GSEA analysis. Differences in hallmark pathway activities between the high- and low-risk groups scored by GSVA **(E)**.Correlation between the HPRGS and hallmark pathway activities scored by GSVA **(F)**. **(G)** The forest map depicted the relationship between hallmark pathway activities scored by GSVA and OS in the TCGA-LIHC cohort.

To further explore the differences in biological functions between patients in high- and low-risk groups, we performed functional enrichment analysis on the DEGs in these two groups. In the gene set enrichment analysis (GSEA) based on the GO gene set, the low-risk group was highly enriched in amino acid catabolism, amino acid metabolism, cellular amino acid catabolism, fatty acid oxidation, and fatty acid catabolism, whereas the high-risk group was highly enriched in adaptive immune response, B cell activation, B cell receptor signaling pathway, cell division, and chromosome segregation (**Figures 5A, B**). The GSEA based on the KEGG gene set showed that the low-risk group had higher activity in complement and coagulation cascades, drug metabolism cytochrome P450, fatty acid metabolism, amino acid metabolism, and fatty acid catabolism, whereas the high-risk group had higher activity in cell adhesion molecules, cell cycle, and DNA replication (**Figures 5C, D**).

Further GSVA analysis based on the hallmark gene set revealed that the high-risk group had higher activity in G2/M checkpoint, E2F transcription factors, mTOR signaling pathway, PI3K-AKT-mTOR signaling pathway, whereas the low-risk group had higher activity in lipogenesis, fatty acid metabolism, bile acid metabolism, and oxidative phosphorylation (**Figure 5E**). Additionally, the correlation analysis between HPRGS and the scores of oncogenic-related hallmarks indicated that HPRGS was closely related to cancer-related biological processes and metabolic pathways (**Figure 5F**). To investigate whether the oncogenic-related hallmark scores were associated with the prognosis of patients with HCC, we performed a survival analysis. The results showed that the pathways positively correlated with HPRGS, such as the G2/M checkpoint, E2F transcription factors, mTOR signaling pathway, and PI3K-AKT-mTOR signaling pathway, were adverse prognostic factors for patients with HCC. In contrast, the pathways negatively correlated with HPRGS, such as lipogenesis, fatty acid metabolism, bile acid metabolism, and oxidative phosphorylation, were associated with good prognosis (all P<0.05, **Figure 5G**). In our study, we found that the biological functions of patients in the high-risk group were mainly enriched in functions and pathways related to cancer development, whereas the biological functions of patients in the low-risk group were mainly enriched in metabolic-related functions and pathways. The activation or inhibition of these pathways may contribute to the different prognostic outcomes observed in the high- and low-risk groups.

### 3.5 Genomic variation landscapes and intratumor heterogeneity in high- and low-risk patient groups

**Figure 6:**
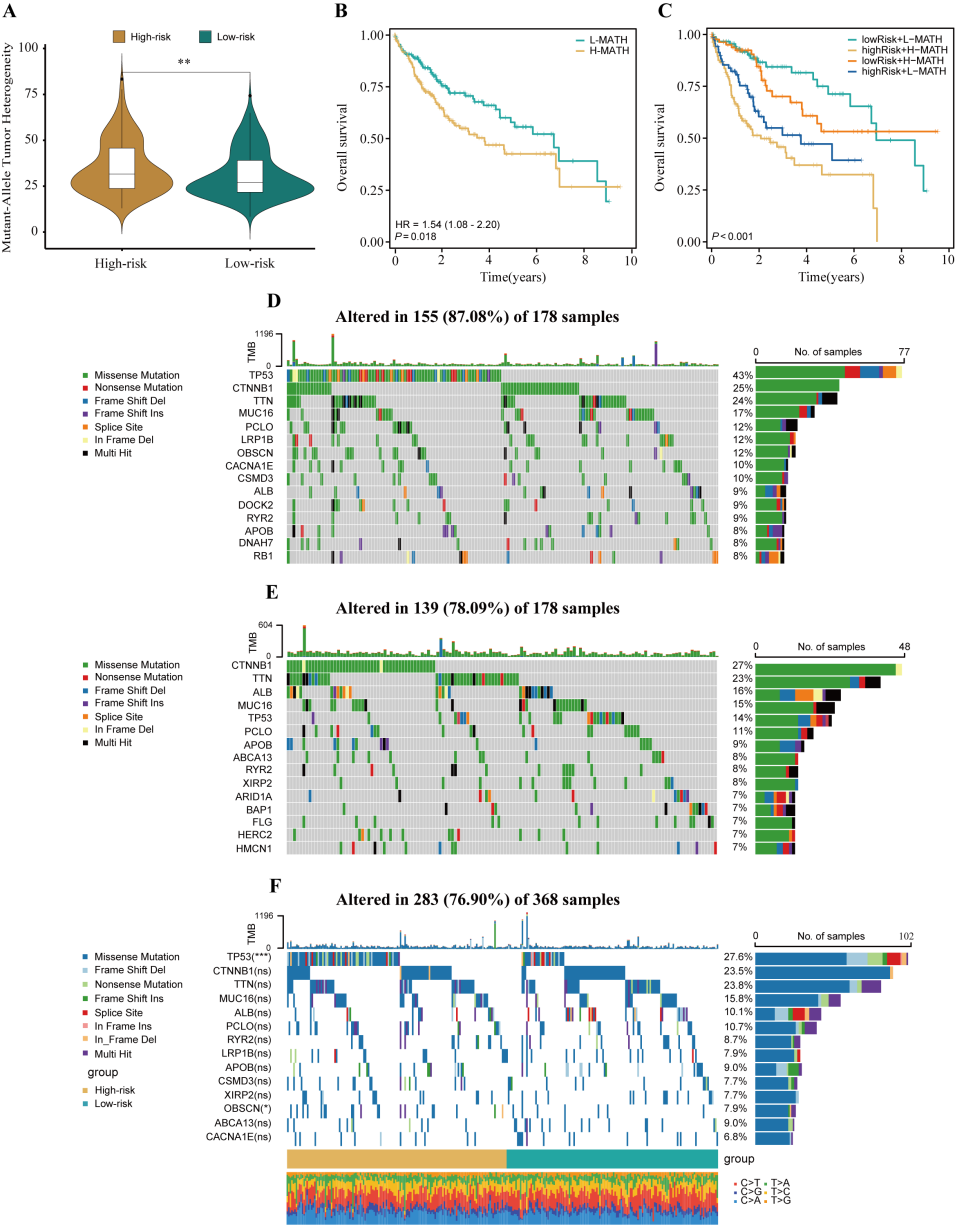
The genomic disparities between high- and low-risk populations. **(A)** Violin plot showed MATH scores between the high- and low-risk groups. **(B)** Kaplan-Meier curve shows the difference in OS between high- and low-MATH score groups. **(C)** Kaplan-Meier curve analysis for OS by combining the MATH score and the HPRGS risk score. The waterfall plot of the somatic mutation landscape in high- **(D)** and low-risk patients **(E)** in the TCGA-LIHC cohort. **(F)** The waterfall plot of the differential somatic mutation landscape in high- and low-risk groups. ns P>0.05, *P < 0.05, **P < 0.01, and ***P < 0.001.

ITH, as a cancer genomic feature, results from the accumulation of gene mutations (42). Studies have confirmed that ITH is positively correlated with chemotherapy resistance in malignant tumors (43). The calculation results showed that in high-risk patients with HCC, the MATH score was relatively high, indicating a more severe degree of ITH (P<0.01, **Figure 6A**). To investigate the relationship between ITH and prognosis in patients with HCC, a survival analysis was conducted. The results showed that the MATH score was positively correlated with poor prognosis in patients with HCC (P = 0.018, HR = 1.54, **Figure 6B**). By combining the MATH score with HPRGS, the prognosis of patients in the “high-risk + high-MATH” group was significantly worse than that of patients in the “low-risk + low-MATH” group (P<0.001, **Figure 6C**).

To explore the differences in genomic mutation frequencies between the high- and low-risk groups, we depicted the mutation landscapes of both groups. The results showed distinct mutation spectra between the high- and low-risk groups (**Figures 6D, E**). As shown in the figure, TP53 was the most common mutated gene in the high-risk group, whereas CTNNB1 was the most common mutated gene in the low-risk group. To further analyze, we combined the top 10 mutated genes in the high- and low-risk groups and conducted a differential analysis to investigate whether there were differences in mutation rates between the two groups. After removing duplicate genes, 14 genes were obtained, among which TP53 and OBSCN had significantly different mutation frequencies between the high- and low-risk groups, with higher mutation frequencies in the high-risk group (both P<0.05, **Figure 6F**).

### 3.6 Patients in high- and low-risk groups have different tumor immune microenvironments

**Figure 7:**
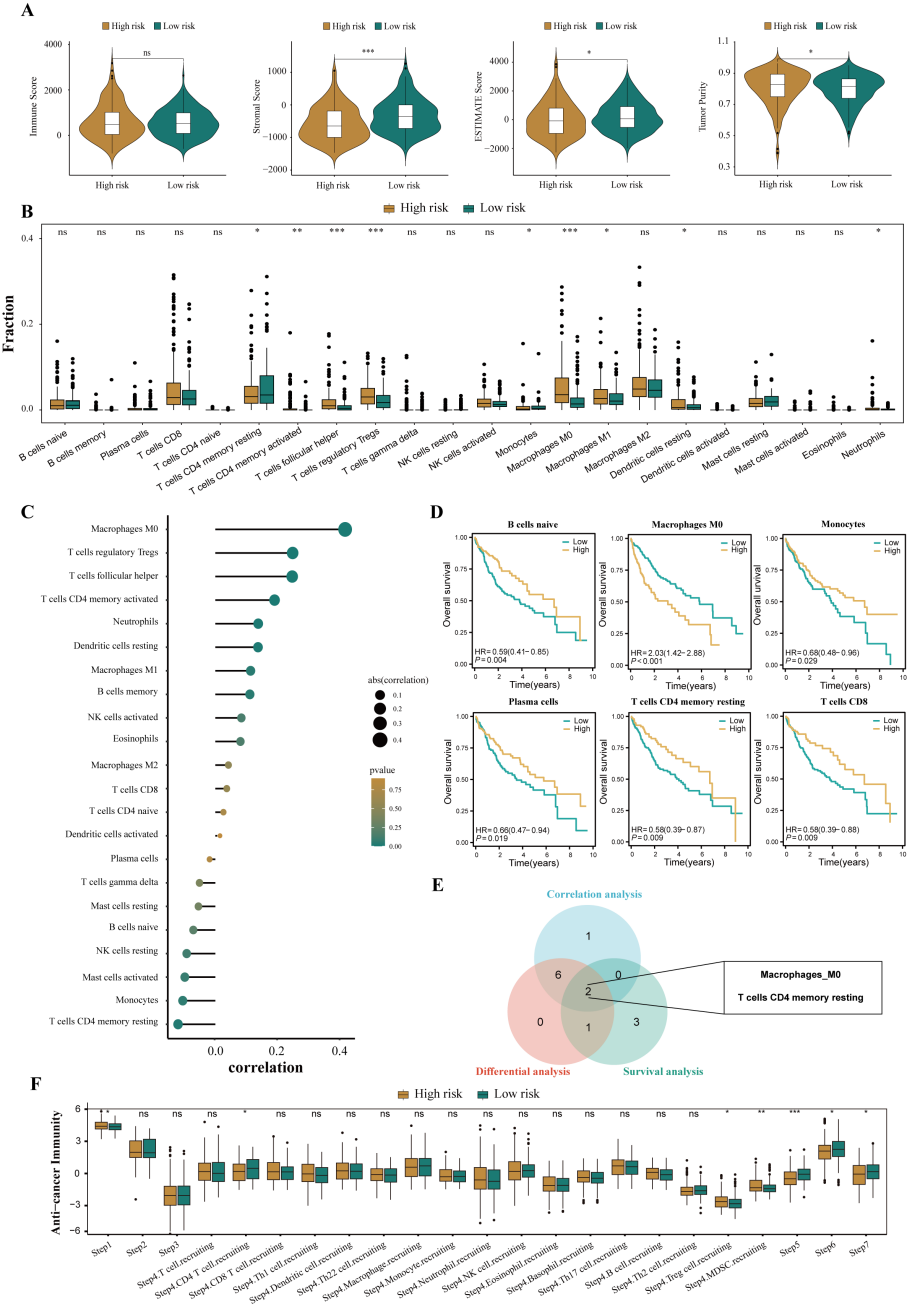
Disparities in the immune microenvironment between high- and low-risk groups. **(A)** The violin plots showed the differential between low- and high-risk groups in immune score, stromal score, the ESTIMATE score, and the tumor purity. **(B)** The abundance of each infiltrated cell type between high- and low-risk groups, quantified by the CIBESORT algorithm. **(C)** Correlation analysis between infiltrated cells and HPRGS. **(D)** Kaplan-Meier curves showed the association between the abundance of 6 infiltrated cell types and OS. **(E)**Venn plot showed the intersecting cell types of differential analysis, correlation analysis, and survival analysis. **(F)** The histogram showed the difference in the seven-step anti-cancer immunity cycle activity between high- and low-risk populations. ns P>0.05, *P < 0.05, **P < 0.01, and ***P < 0.001.

To assess the immune infiltration status of patients with HCC, this study used the ESTIMATE algorithm to calculate the immune score, stromal score, comprehensive score, and tumor purity score of the high- and low-risk groups. The results showed that the high-risk group performed poorly in the comprehensive score of the microenvironment, whereas the tumor purity score was relatively high (all P<0.05, **Figure 7A**). To further explore the differences in specific immune cell infiltration between high- and low-risk groups, we quantitatively analyzed the abundance of immune cell infiltration in each sample using the CIBERSORT deconvolution algorithm. The results showed that the tumor immune microenvironment of patients in the high-risk group was rich in regulatory T cells, M0 macrophages, activated memory CD4+ T cells, follicular helper T cells, M1 macrophages, and neutrophil infiltration. In contrast, the tumor immune microenvironment of patients in the low-risk group was rich in resting memory CD4+ T cells and monocytes (all P<0.05, **Figure 7B**). The correlation analysis results were also similar (**Figure 7C**). In addition, we explored the relationship between different tumor microenvironment (TME) cell types and OS in patients with HCC. The results showed that the infiltration abundance of six cell types was correlated with the prognosis of patients with HCC (all P<0.05, **Figure 7D**). Combining the results of differential analysis, correlation analysis, and survival analysis, we finally identified two types of intersecting cells, namely, resting memory CD4+ T cells and M0 macrophages (**Figure 7E**). This may mean that the infiltration of these two immune cells is of significant importance in the prognosis and development of HCC.

Given the complexity of intratumoral immune responses and the microenvironment, the degree of immune cell infiltration alone cannot fully reflect immune activation and exhaustion. By assessing the activity of various aspects of the anti-cancer immune cycle, a deeper understanding of the role of immune cells in the anti-tumor process can be achieved, thereby improving the accuracy of immunotherapy guidance [49]. Calculations revealed that the high-risk group performed more prominently in antigen release from cancer cells, recruitment of regulatory T cells, infiltration of tumor-suppressing myeloid cells derived from bone marrow, recognition of cancer cells by T cells, and cancer cell killing (all P<0.05, **Figure 7F**). These results suggest that the high-risk group may exhibit a reduced anti-cancer efficacy in the immune cell function cycle compared to the low-risk group.

### 3.7 Evaluation and prediction of HCC treatment strategies

To explore the application of HPRGS in predicting the treatment response of HCC, including tumor volume doubling time (TVDT), response to transarterial chemoembolization (TACE) treatment, and prediction of immune and targeted drug treatment responses, potential therapeutic drugs for patients in the high-risk group were explored.

#### 3.7.1 Prediction of the response to transarterial chemoembolization

**Figure 8:**
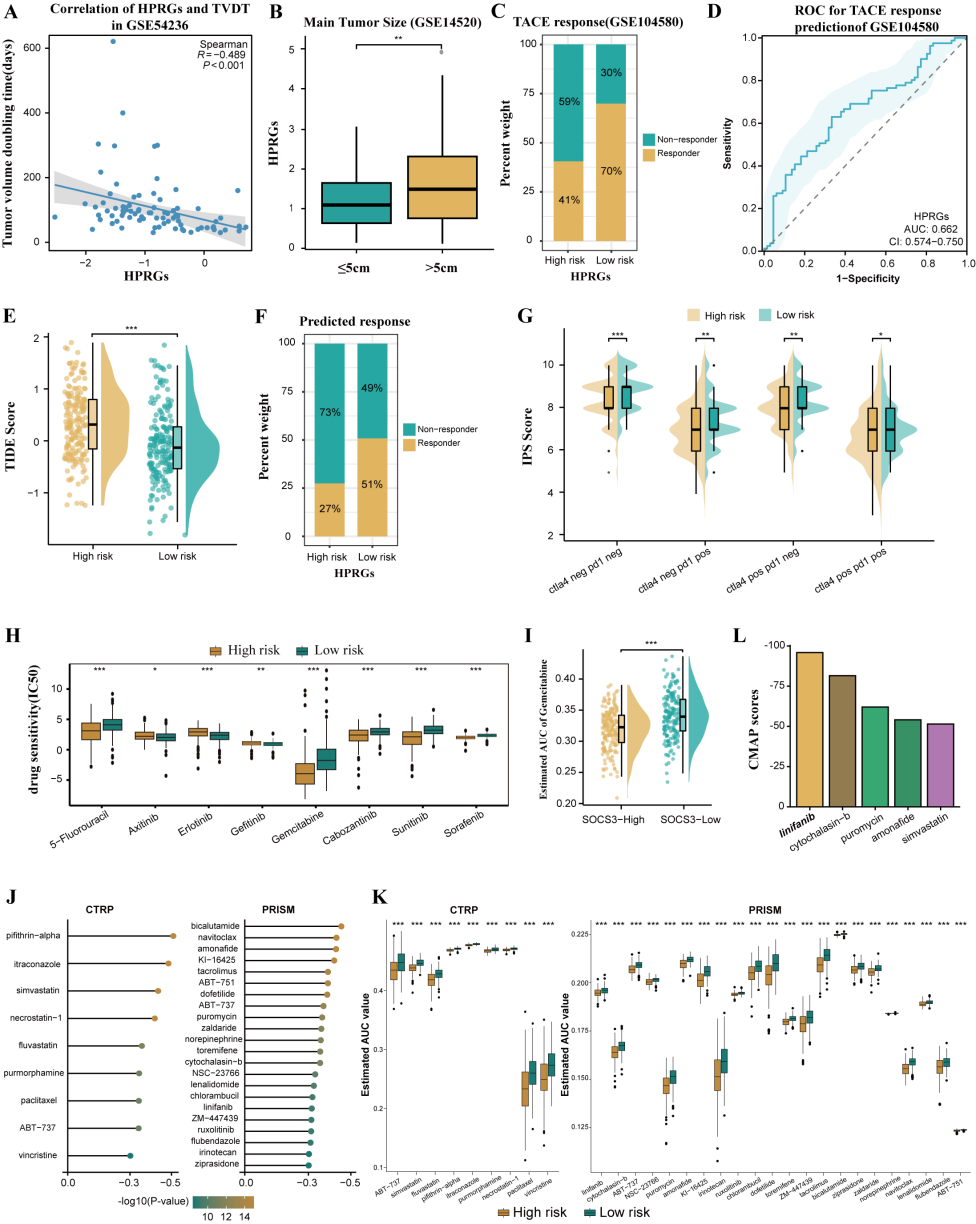
Differences in benefit from different treatment options in the high- and low- risk groups. **(A)** Correlation between HPRGS score and TVDT in GSE54236. **(B)** The boxplot showing the difference in main tumor size between high- and low-risk populations in GSE14520. **(C)** Comparing TACE treatment response rates between high- and low-risk populations in GSE104580. **(D)** ROC curve to predict TACE treatment response using the HPRGS score. **(E)**The cloud rain plot showed the difference in TIDE scores between high- and low-risk groups. **(F)** Comparing the immunotherapy response rate predicted by TIDE algorithm between high- and low-risk populations. **(G)** The violin plots showed the difference in IPS scores between high- and low-risk groups. **(H)** Sensitivity comparison of small molecule drugs in high - and low-risk groups. **(I)** Comparison of estimated gemcitabine’s sensitivity between high and low SOCS3 expression groups. **(J)** Barplot of CMap scores for the top 5 drugs in the high-risk group. **(K)**The results of Spearman’s correlation analysis of CTRP-derived compounds and PRISM-derived compounds. **(L)**The results of differential drug response analysis of CTRP-derived compounds and PRISM-derived compounds, the lower values on the y-axis of boxplots imply greater drug sensitivity. *P < 0.05, **P < 0.01, and ***P < 0.001.

In the GSE54236 cohort, we observed a negative correlation between HPRGS and TVDT (R = -0.489, P < 0.001, **Figure 8A**). Additionally, in the GSE14520 cohort, a correlation between HCC and tumor size was further confirmed, with higher HPRGS observed in patients with larger tumors compared to those with smaller tumors (P< 0.01, **Figure 8B**). Subsequently, HPRGS was used for validation in the GSE104580 cohort, and the results showed that the proportion of responders was higher in the low-risk group than in the high-risk group (70% vs. 41%, **Figure 8C**). Furthermore, the AUC value for predicting the TACE treatment response rate in patients with HCC using HPRGS was 0.662 (CI: 0.574–0.750, **Figure 8D**). Therefore, HPRGS can be used to predict the efficacy of TACE treatment in patients with HCC.

#### 3.7.2 Prediction of immunotherapy sensitivity

To predict the sensitivity of patients with HCC in the high and low-risk groups to immunotherapy, patients in the low-risk group exhibited higher TIDE scores and a higher immunotherapy response rate. This suggests that patients in the low-risk group may benefit more from ICI treatment (P< 0.001, **Figures 8E, F**). Further IPS scoring analysis revealed that compared to the high-risk group, the low-risk group had significantly higher IPS scores for PD-1 and CTLA4 inhibitors, indicating that patients in the low-risk group were more sensitive to PD-1 and CTLA4 inhibitor treatments than those in the high-risk group (both P< 0.05, **Figure 8G**). In summary, patients in the low-risk group are more likely to benefit from immunotherapy.

#### 3.7.3 Drug sensitivity analysis

Drug resistance is currently a major cause of poor prognosis in tumors, and the emergence of drug resistance seems to be an inevitable consequence of tumor exposure to kinase-targeted therapy (44). Therefore, we used the GDSC database to predict drug sensitivity in patients with HCC with different HPRGS. The results showed that the IC50 of 5-fluorouracil, gemcitabine, sorafenib, cabozantinib, and sunitinib was significantly lower in the high-risk group, whereas the IC50 of axitinib, erlotinib, and gefitinib was significantly lower in the low-risk group (all P< 0.05, **Figure 8H**). These findings suggest that patients in the low-risk group may respond better to axitinib, erlotinib, and gefitinib treatment, whereas patients in the high-risk group may be more sensitive to 5-fluorouracil, gemcitabine, sorafenib, cabozantinib, and sunitinib.

#### 3.7.4 Exploration of potential drugs for high-risk group patients

To explore potential therapeutic drugs for high-risk patients with HCC, we analyzed data based on the CTRP database and the relative inhibition database in PRISM (45). To ensure the reliability of our plan, gemcitabine was used as a reference drug to study whether the estimated sensitivity was consistent with clinical practice. An experimental study showed that increased resistance to gemcitabine in HCC was associated with decreased SOCS3 expression, whereas increased SOCS3 expression could inhibit resistance to gemcitabine in HCC (45). Consistent with this study, our results confirmed that patients with higher SOCS3 expression levels had significantly lower predicted AUC values, indicating higher sensitivity to gemcitabine (P < 0.001, **Figure 8I**). Next, we used this formula to identify potentially sensitive drugs for high-risk group patients and screened out 31 drugs in CTRP and PRISM. The predicted AUC values of these drugs were statistically negatively correlated with HPRGS and were significantly lower in the high-risk group (all R < -0.3, **Figure 8J**). In addition, based on the difference analysis between the high- and low-risk groups of HCC (all P < 0.001, **Figure 8K**), we further applied the CMap tool to determine the candidate compounds of HCC. After cross-analysis of the results obtained from CTRP and PRISM, we finally screened out five potential candidate compounds: the tyrosine kinase inhibitor linifanib, cytochalasin B, puromycin, amifetipine, and simvastatin. Among them, linifanib exhibited high sensitivity in the high-risk patient population with a CMap score of -95.88, indicating that it may be a potential therapeutic drug for high-risk group patients (**Figure 8L**).

### 3.8 Construction and evaluation of nomogram

**Figure 9:**
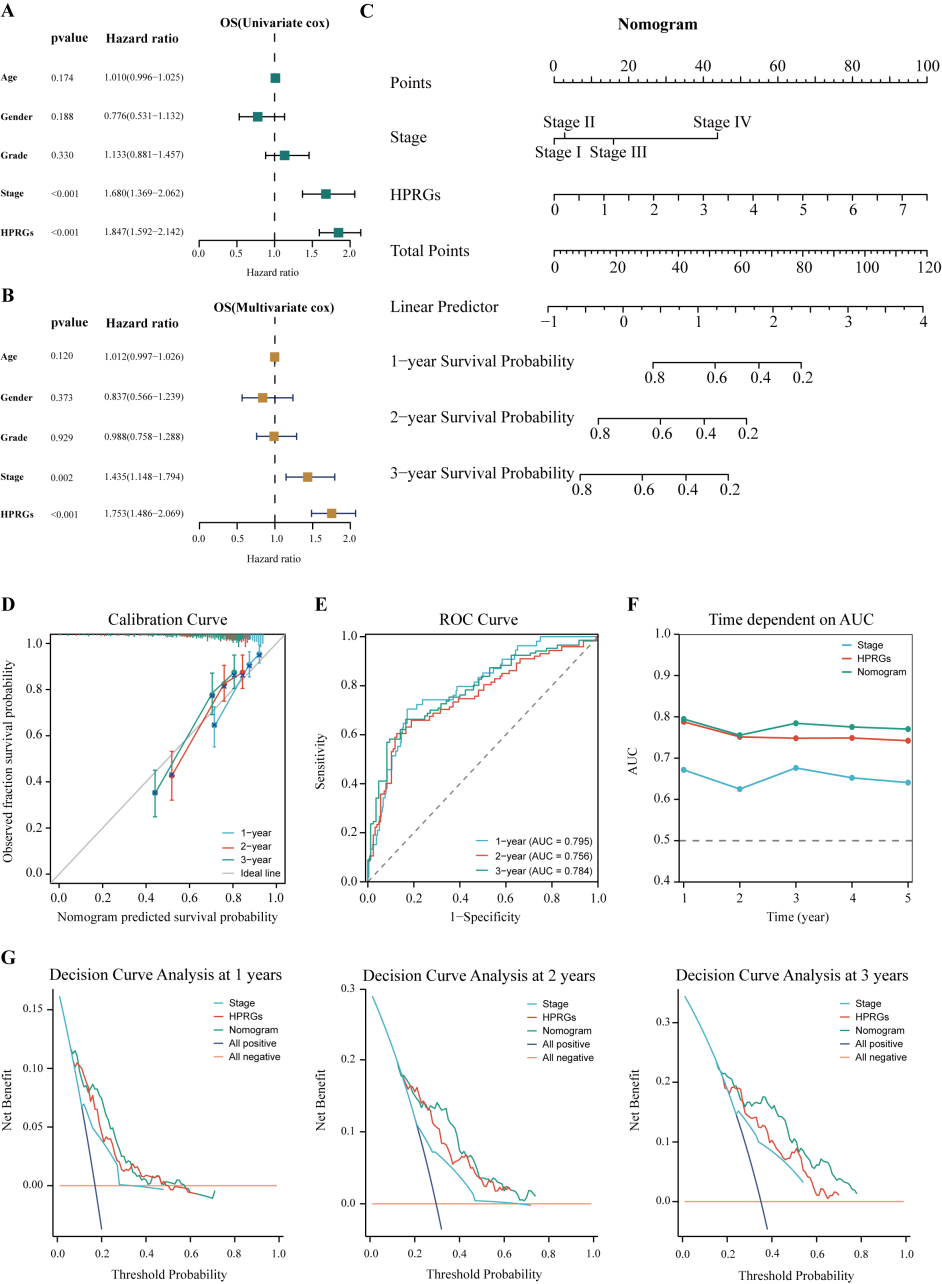
Establishment and verification of the nomogram. Univariate **(A)** and multivariate **(B)** analyses of the clinical characteristics and HPRGS for the OS in the TCGA-LIHC cohort. **(C)** Construction of the nomogram based on the HPRGS and independent prognostic clinical characteristics. **(D)** Calibration curve of the nomogram for 1-, 2-, and 3-year OS. **(E)** ROC curves showed the prediction performance of the nomogram in 1-, 2-, and 3-year OS. **(F)** The comparison of the AUC between the nomogram and other clinical characteristics. **(G)** Decision curve analysis showed net benefits by applying a nomogram and other clinical features at 1-, 2-, and 3-year OS.

To enhance the clinical utility of HPRGS, univariate and multivariate Cox regression analysis was performed on patients with HCC in TCGA-LIHC cohort. The results showed that in univariate analysis, HPRGS were independent prognostic factors for OS (HR>1, P<0.001, **Figure 9A**). In multivariate analysis, HPRGS remained independent prognostic factors for OS (HR>1, P<0.001, **Figure 9B**), indicating that HPRGS have reliable prognostic evaluation ability in patients with HCC. We constructed a nomogram by combining the HPRGS and independent prognostic clinicopathological characteristics (**Figure 9C**).

The calibration curve showed excellent consistency between the nomogram predictions and actual observations (**Figure 9D**). ROC curve analysis showed that the AUC values of the nomogram at 1-, 2-, and 3-years were 0.814, 0.760, and 0.788, respectively, confirming its high prediction accuracy (**Figure 9E**). In addition, the multi-index AUC curve graph confirmed the stability and robustness of the nomogram, which was superior to other clinical characteristics in predicting 1- to 5-year survival rates (**Figure 9F**). Decision curve analysis at 1-, 2-, and 3-years showed that the nomogram had better net clinical benefit compared to other clinical characteristics (**Figure 9G**). These findings revealed that the nomogram can provide reliable and accurate evidence for personalized prognosis prediction in HCC.

### 3.9 Verification of HPRGS in HCC cDNA microarray cohorts

**Figure 10:**
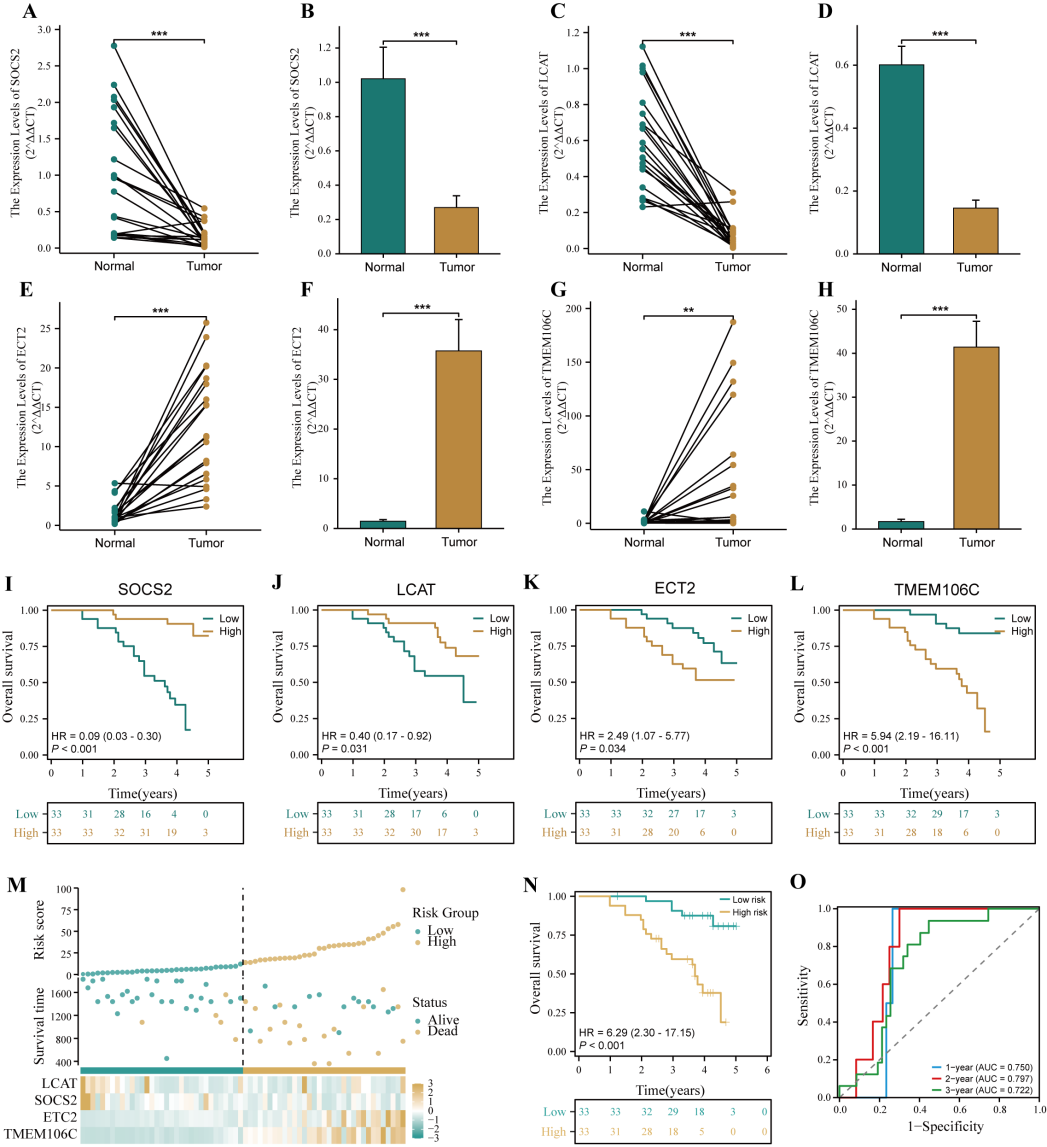
Verification of HPRGS by qRT-PCR in HCC cDNA microarray cohorts. Differential expression of SOCS2 among paired samples **(A)** and unpaired samples **(B)** in HCC cDNA microarray by qRT-PCR. Differential expression of LCAT among paired samples **(C)** and unpaired samples **(D)** in HCC cDNA microarray by qRT-PCR. Differential expression of ECT2 among paired samples **(E)** and unpaired samples **(F)** in HCC cDNA microarray by qRT-PCR. Differential expression of TMEM106C among paired samples **(G)** and unpaired samples **(H)** in HCC cDNA microarray by qRT-PCR. The Kaplan–Meier curves showed the prognosis of the patients grouped by the median expression value of SOCS2 **(I)**, LCAT **(J)**, ECT2 **(K)**, TMEM106C **(L)** in HCC cDNA microarray cohorts. **(M)** The risk factor plot of the HCC cDNA microarray cohorts. **(N)** The Kaplan–Meier curves of OS according to the HPRGS in the HCC cDNA microarray cohort. **(O)** ROC curves showed the specificity and sensitivity of HPRGS and clinical characteristics in predicting 1-, 2-, and 3-year OS in the HCC cDNA microarray cohort. **P < 0.01 and ***P < 0.001.

To verify the accuracy of HPRGS in the real world, we further validated HPRGS in HCC cDNA microarray cohorts using qRT-PCR. Firstly, the results of the differential analysis showed that SOSC2 and LCAT genes were highly expressed in normal tissues, and ECT2 and TMEM106C were overexpressed in tumor tissues, regardless of paired or unpaired samples (all P<0.05, **Figures 10A–H**). Then, K-M curve analysis showed that SOSC2 and LCAT were positively correlated with good prognosis, whereas ECT2 and TMEM106C were positively correlated with poor prognosis, which was consistent with previous findings (all P<0.05, **Figures 10I–L**). Finally, patients were divided into high- and low-risk groups by using the median risk score. The results showed that HPRGS were positively correlated with poor prognosis in HCC cDNA microarray cohorts (P<0.05, **Figures 10M, N**), and 1-, 2-, and 3-year ROC curves exhibited excellent predictive efficacy (the AUCs of HPRGS were 0.750, 0.797, and 0.722 at 1-, 2-, and 3-years, respectively; **Figure 10O**).

## 4 Discussion

In this study, we used a new computing framework to identify stable and reliable prognostic features. Through validation, the HPRGS we constructed has good predictive performance. In addition, we explained the potential reasons for the prognostic differences in different HPRGS groups through mutation, immune infiltration, and functions analysis, and guided clinical diagnosis and treatment through drug sensitivity analysis and the construction of nomograms.

The framework includes 10 machine learning algorithms and 101 combinations (46). We used this framework to screen genes and construct a prognostic model with high predictive accuracy and interpretability through multivariable Cox regression analysis. In recent years, with the advancement of high-throughput sequencing technologies, the development of cancer prediction models based on gene expression has become a significant research focus. Notably, models such as the prognostic signature constructed by Tang et al. based on genes associated with aging (47), the prognostic signature by Chen et al. based on features regulating glycosylation (48), and the prognostic signature by Guo et al. related to genes associated with the heterogeneity of NK cells (49), have all demonstrated excellent predictive efficacy. However, most current research is predicated on specific gene sets (50, 51), often overlooking the roles of other genes outside these defined sets. Machine learning can leverage its capacity for dimensionality reduction and variable selection in large datasets to identify and incorporate the most critical variables in model construction. Additionally, in this study, we compared our recently published models and found that our HPRGS has a favorable prognostic predictive role for HCC patients. Using survival analysis and single and multivariable Cox analysis, we found that HPRGS can stratify the risk of patients with HCC in terms of OS and are independent prognostic factors. In addition, the predictive accuracy of HPRGS is significantly better than other clinical features. The stability of prognostic stratification among clinical subgroups further confirms the robustness of HPRGS.

In our study, the biological functions of patients with HCC in the high-risk group were mainly enriched in functions and pathways related to cancer development, such as cell division, cell adhesion molecules, cell cycle, and DNA replication. Conversely, the biological functions of patients with HCC in the low-risk group were mainly enriched in metabolism-related functions and pathways. The activation or inhibition of these pathways may affect the different prognostic outcomes observed in the high- and low-risk groups. In addition, the hallmark pathway positively correlated with HPRGS is considered to be an oncogenic pathway, including the MYC and PI3K-AKT-mTOR signaling pathways. As mentioned earlier, these pathways show abnormal hyperactivation in various types of cancer. This abnormal hyperactivation has been shown to drive cancer cell proliferation, invasion, and metastasis, and is usually associated with poor clinical prognosis (52, 53). We explored the mutation spectrum and ITH of patients in different high- and low-risk groups. Previous studies have shown that the higher the degree of ITH, the higher the possibility of tumor infiltration and drug resistance (54). This finding is consistent with our observations that patients in the high-risk group have relatively higher drug resistance and poor clinical prognosis compared to the low-risk group.

In the high-risk group, the mutation rates of TP53 and OBSCN genes increased significantly. TP53 is a well-known tumor suppressor gene, and its mutation is closely related to the poor prognosis of HCC (33). An increasing number of studies have confirmed that p53 has a significant impact on the metabolism of normal cells and cancer cells. In tumor cells, mutant p53 can positively regulate glycolysis, whereas negatively regulates cell production, tricarboxylic acid cycle, and lipid metabolism (55, 56). In addition, a large number of copy number changes and mutations have been observed in the OBSCN gene in many cancer types. Some studies on this gene have also demonstrated that the decrease or alteration of OBSCN gene expression largely disrupts cell integration and activates the occurrence of cancer; furthermore, several studies on OBSCN gene mutations have revealed its potential role in melanoma, glioblastoma, colorectal cancer, lung cancer, breast cancer, and pancreatic cancer. Therefore, the OBSCN gene may have the characteristics of a tumor suppressor gene and can prevent cell transformation (57–59). Further studies have explored the immune microenvironment of patients with HCC in the high- and low-risk groups and the results showed that the high-risk group had more infiltrating regulatory T cells, but there was higher infiltration of resting memory CD4+ T cells in the low-risk group. In addition, through the analysis of immune function and anti-tumor immune cycle, the immune effector cell activity of the high-risk group was lower, suggesting that the TME of the high-risk group patients may be in a suppressed state.

HCC exhibits significant heterogeneity in molecular characteristics and biological behavior, posing significant challenges for clinicians in managing cancer patients (60). Therefore, it is crucial to predict the best treatment strategy before treatment to improve patient prognosis and minimize treatment-related costs. Hence, there is an urgent need to optimize personalized treatment plans for HCC. The HPRGS developed in this study can predict the efficacy of TACE treatment for patients with HCC. Although TACE is considered the preferred treatment for patients with intermediate-stage HCC, studies have shown that its ORR is only 52.5% (61). Therefore, it is particularly important to seek better predictors of TACE treatment response. Previous studies have revealed that patients with shorter TVDT often have lower survival rates, increased risk of recurrence, and poor response to TACE treatment (62, 63). Accurate prediction of TVDT can help avoid overdiagnosis and overtreatment, reduce economic losses, and improve patient quality of life without negatively affecting prognosis (64). In this study, patients in the high-risk group had shorter TVDT and lower response rates to TACE treatment compared to those in the low-risk group. This finding is consistent with previous studies. ICI has brought significant survival benefits to cancer patients by activating the immune system to eliminate cancer cells (65). However, its clinical application is limited by its low response rate in cancer treatment (66). This study used TIDE and IPS algorithms to predict that patients in the low-risk group had lower TIDE scores, higher immunotherapy response rates, and higher IPS scores. This suggests that patients in the low-risk group may exhibit better outcomes in receiving ICI treatment compared to those in the high-risk group. Furthermore, we predicted the sensitivity of small-molecule drugs in the treatment of HCC in both high- and low-risk groups. The results showed significant differences in IC50 values between the two groups, which may help improve the precision of treatment plans and achieve more effective liver cancer treatment. Interestingly, the high-risk group exhibits increased sensitivity to 5-fluorouracil and gemcitabine, two drugs that affect DNA synthesis, which may be related to the significant activation of pathways such as the cell cycle and DNA replication in the high-risk group. Because the above studies suggest that patients in the low-risk group may benefit more from immunotherapy, we integrated the results of CTRP, PRISM, and CMap to specifically identify drugs that may be effective for patients in the high-risk group (33–35). Finally, we identified the tyrosine kinase inhibitor lenvatinib as a potential drug for patients in the high-risk group. Lenvatinib (ABT-869) is a tyrosine kinase inhibitor whose anti-angiogenic activity has been explored in many clinical trials (67). Given its potential efficacy in the HPRGS high-risk group of patients with HCC, this finding may provide a reference for future research. To provide a convenient tool for quantifying HCC survival analysis, we constructed a nomogram that integrates HPRGS and independent prognostic clinical features. The nomogram exhibits good discrimination, and ROC curves, C-indices, and calibration curves indicate its high predictive accuracy. Decision curve analysis showed that the nomogram outperforms other clinical features in terms of net clinical benefit.

Although our study has yielded promising results, several limitations should be acknowledged. First, the limited clinical data available in the public cohort may have masked potential associations between HPRGS and certain clinical variables. Therefore, it is necessary to conduct more comprehensive and standardized data collection to further explore the clinical value of HPRGS. Second, although we evaluated and validated the HPRGS in training and validation cohorts, large-scale, multicenter prospective studies are needed to further confirm our findings. In addition, *in vitro* and *in vivo* studies are required to reveal the biological functions of HPRGS-related genes in HCC. Finally, although we predicted the sensitivity of high- and low-risk groups to various small-molecule drugs, our predictions need to be validated through *in vitro* drug screening and clinical trials. Despite these limitations, our findings provide useful insights for risk assessment and precision medicine treatment of HCC and lay a foundation for further research in this area.

## 5 Conclusions

In conclusion, our study presents the development and validation of the HPRGS, offering a potent tool for predicting survival outcomes and treatment responses in HCC patients. The signature’s ability to delineate distinct subgroups with unique pathway activities and tumor microenvironments provides insights into HCC heterogeneity. Moreover, our exploration of potential therapeutic agents for high-risk patients, aiming to improve prognoses and refine treatment strategies in HCC management.

## Data Availability

The original contributions presented in the study are included in the article/**Supplementary Materials**. Further inquiries can be directed to the corresponding authors.
