# Supplementary material for: Novel prognostic signature for hepatocellular carcinoma using a comprehensive machine learning framework to predict prognosis and guide treatment
Source: Front Immunol. 2024 Sep 24;15:1454977. doi: 10.3389/fimmu.2024.1454977 (PMC11458406; doi:10.3389/fimmu.2024.1454977)
Supplement: Supplementary file 1 [file DataSheet1.docx]

Supplementary Material

**Supplementary Table**

**Supplementary table 1.** Results of differentially expressed genes across various datasets.

**Supplementary Figure**


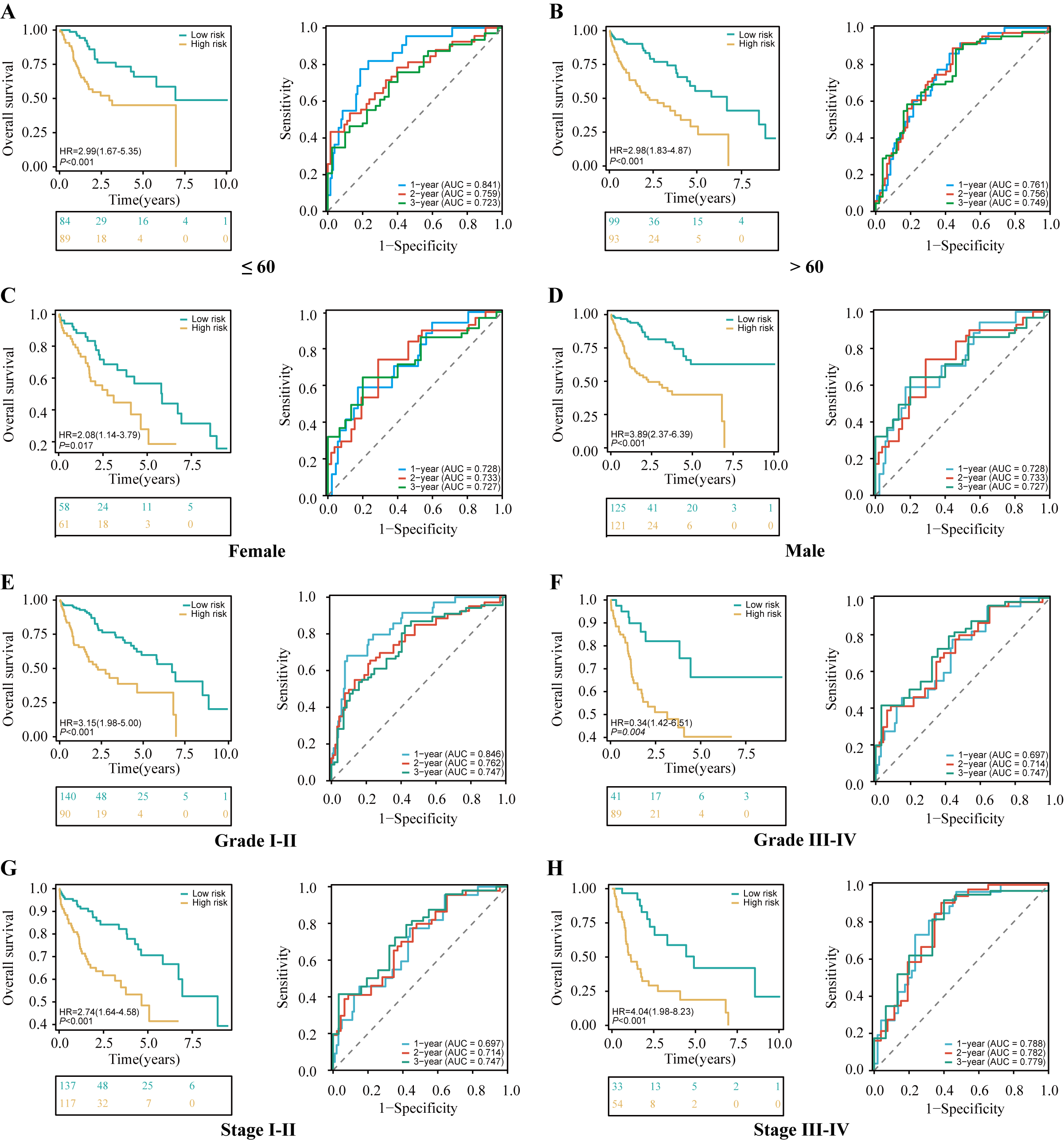


**Supplementary Figure 1.** The application of subgroup analysis in clinical characteristics. Comparison of overall survival and time-dependent ROC curves for LIHC patients of different ages (A, B), sexes (C, D), grade(E, F) and pathological stages(G, H) between the high-risk and low-risk groups.
